# HIV Care for Adolescents and Young Adults: Comparing Nurse Practitioner and Physician Care in Engagement, Trust, and Clinical Outcomes

**DOI:** 10.21203/rs.3.rs-8485057/v1

**Published:** 2026-01-06

**Authors:** Emily Anne Barr, Suzanne E. Courtwright, Sheryl Malone-Thomas, Lori Silveira, Deborah Kacanek, Paul Cook, Sean M. Reed, Hillary Dunlevy

**Affiliations:** University at Buffalo School of Nursing; Columbia University School of Nursing; Cizik School of Nursing at UT Health at Houston; University of Colorado School of Medicine; Harvard T.H. Chan School of Public Health; University of Colorado College of Nursing; University of Colorado College of Nursing; University of Colorado School of Medicine

**Keywords:** HIV, youth, nurse practitioners, task-shifting, trust, telehealth, global health workforce

## Abstract

**Background:**

Shortages in the HIV workforce threaten equitable access to care for youth with HIV (YWH) experiencing high rates of undiagnosed infection and suboptimal engagement. Nurse practitioners (NPs) may expand HIV care capacity, but evidence is limited.

**Methods:**

We conducted a cross-sectional study in two U.S. adolescent and adult HIV programs. Electronic health record data were merged with patient-reported surveys assessing trust, adherence, and stigma across an eight-month pre– and post–COVID-19 period. Provider type’s (NP vs. physician) were compared.

**Results:**

Among 109 participants (mean age 26 years), viral suppression did not differ by provider type. Youth receiving NP-led care had higher CD4 counts, more visits, greater telehealth use, and were more likely to report higher patient-provider trust.

**Conclusions:**

NP-led HIV care for youth achieved clinical outcomes comparable to physician care and was associated with higher trust and engagement, underscoring the role of nurse practitioners in strengthening the HIV workforce.

## Introduction

A shortage in the U.S. HIV clinical provider workforce limits equitable access to HIV prevention and treatment services, particularly for adolescents and young adults aged 13–34, who account for 58% of HIV infections nationwide (Armstrong, 2020; Bono et al., 2021; Centers for Disease Control and Prevention, 2023; Kelly et al., 2024). Predicted more than a decade ago, this shortage reflects the retirement of early HIV specialists, limited growth in infectious diseases training programs, and insufficient HIV-focused pathways in primary care residencies (Budak et al., 2021; Gilman et al., 2016; Gilman B., 2013; Stevens et al., 2008; Weiser et al., 2016; Weiser et al., 2019). Despite efforts to expand training, infectious disease fellowship enrollment declined by 8.1% between 2008 and 2022, and more than half of fellowship programs did not fill available positions in 2024 (Kelly et al., 2024). In 2024, 51% of fellowship programs did not fill available spots for infectious diseases fellows. Further, dedicated HIV training pathways built into family medicine residency programs generated only 38% of physicians providing HIV health services (Budak et al., 2021). Compounding these challenges, few HIV-trained clinicians practice in southern or rural regions, which now represent the epicenter of the U.S. HIV epidemic (Centers for Disease Control and Prevention, 2023; Schafer et al., 2017).

National workforce projections further underscore these inequities. Health Resources and Services Administration analyses estimate that the rural infectious disease workforce will meet only 14% of projected demand between 2024 and 2036 (Department of Health and Human Services, 2024). Similarly, the adolescent medicine workforce, another critical pipeline for youth HIV prevention and treatment, is projected to decline and remains unevenly distributed geographically (Fields et al., 2024). Insurance access further constrains care delivery, as an estimated 25% of family physicians and 15% of pediatric primary care physicians caring for youth with HIV no longer accept Medicaid, the primary payer for people living with HIV (Kates et al., 2021). Together, these factors create persistent barriers to youth-focused HIV prevention and treatment and threaten progress toward the Ending the HIV Epidemic initiative, which aims to reduce new HIV infections in the United States by 2030 (Armstrong, 2020; HHS, 2022).

These workforce gaps are particularly concerning given that youth aged 13–24 have the highest proportion of undiagnosed HIV infections at 44%, substantially exceeding other age groups (Centers for Disease Control and Prevention., 2021). HIV testing rates in this population remain low, with fewer than one in four youth reporting having been tested (Mustanski et al., 2020; Zapata et al., 2024). Evidence indicates that youth are significantly more likely to undergo HIV testing when they have clinicians with whom they can discuss sexual health and HIV openly, underscoring the importance of accessible, developmentally responsive care (Mustanski et al., 2020; Zapata et al., 2024). Ensuring a workforce capable of engaging youth in these conversations is therefore central to HIV prevention and early treatment efforts.

Nurse practitioners represent a rapidly expanding segment of the U.S. healthcare workforce and are well positioned to address these gaps (Bureau of Labor Statistics, 2023; K., 2023). As nationally board-certified advanced practice nurses, NPs receive graduate-level training in advanced assessment, pharmacology, and population-focused care, with an emphasis on addressing social, behavioral, and structural determinants of health Federal investments in HIV-focused NP training programs, including those supported by HRSA, have further strengthened this workforce (Farley et al., 2016). Prior studies demonstrate that HIV care delivered by NPs achieves clinical outcomes comparable to physician-led care, with some evidence of advantages in prevention counseling, adherence support, and retention (Wilson et al., 2005; Zhang et al., 2020; Weiser et al., 2024). However, evidence specific to adolescents and young adults with HIV remains limited, despite their disproportionate burden of disease and elevated risk of disengagement from care (Mgbako et al., 2022). Relational aspects of care, including trust, may be especially salient for sustaining engagement in this population.

This study was conceptually informed by the Trust-Link Relational Transition Model (Blinded, 2025), which frames trust as a relational process that shapes engagement, continuity, and health outcomes for youth with chronic illness. The model integrates nursing and trust theory to emphasize informed trust, relational presence, and collaborative care as mechanisms that support youth engagement during vulnerable care transitions. Given evidence that trust mediates retention and adherence in HIV care, particularly among youth facing stigma and developmental transitions, this framework guided our selection of patient-reported trust measures and our hypothesis that NP-led HIV care would demonstrate relational strengths alongside equivalent clinical outcomes.

Prior studies comparing nurse practitioner and physician HIV care demonstrate comparable quality across key clinical outcomes, including antiretroviral therapy initiation and prescribing, immune markers, viral suppression, and sexually transmitted infection testing (Wilson et al., 2005; Zhang et al., 2020). More recent findings further support the role of nurse practitioners in high-quality HIV care. An analysis of the Centers for Disease Control and Prevention’s Medical Monitoring Project (2019–2021) involving 6,323 adults, found that individuals whose primary HIV care providers were NPs had significantly higher rates of retention in care and were more likely to receive HIV testing recommendations than those with infectious disease (ID) physician providers (Weiser et al., 2024). In addition, among young adults, nurse practitioners provided a greater number of HIV prevention and treatment services per patient than both infectious disease and non–infectious disease physicians, including routine HIV care, STI testing, and adherence counseling (Weiser et al., 2024; Zhang et al., 2020). Despite this growing evidence base, data specific to adolescents and young adults with HIV remain limited (Mgbako et al., 2022). Accordingly, the aim of this study was to compare HIV-specific clinical outcomes and patient-reported outcomes among youth with HIV receiving care from nurse practitioners versus physician providers.

## Methods

This cross-sectional observational study examined clinical characteristics and health outcomes among YWH based on their primary provider type (nurse practitioner [NP] versus physician). Data were abstracted from electronic medical records and included demographics, HIV-specific outcomes, visit types, and engagement in care (EIC). The HIV-specific outcomes of interest were HIV RNA PCR (copies/μL), CD4 absolute cell counts, and CD4 percentage. For each participant, HIV viral suppression was assessed using their HIV RNA PCR test results across the 16-month study period. Participants were classified as “suppressed” if all HIV RNA PCR results during that period were < 200 copies/μL. This binary outcome was used to compare viral suppression across provider types.

Visit types were categorized as in-person, telehealth (video or phone), or hybrid. Engagement in care (EIC) was operationalized as a binary variable. Participants were classified as “engaged in care” using a modified definition based on Ryan White HIV/AIDS Program retention measures and CDC Medical Monitoring Project criteria. Because data were pulled 8 months after the onset of the COVID-19 pandemic (March 15, 2020), the 16-month study period included the 8 months before and after that date. Participants were considered engaged if they had at least one HIV care visit in both time periods, had at least one HIV RNA PCR test during the study window, and demonstrated sustained viral suppression (defined as all HIV RNA PCR results < 200 copies/μL) (Dasgupta et al., 2021; HRSA, 2019). Those not meeting these criteria were classified as “not engaged in care.”

Patient-reported outcomes were collected using a self-administered survey and included measures of patient-provider trust, ART adherence, and HIV stigma. Trust was assessed using three validated tools: the Healthcare Relationship Trust Scale-Revised (HCR Trust Scale-R) (Bova et al., 2012), the Watson Patient Caring Score (WPCS) (Brewer & Watson, 2015), and the Patient Trust Assessment Tool (PaTAT) (Velsen et al., 2017). The HCR Trust Scale-R is a 13-item scale measuring perceived provider trust using a 5-point Likert scale, with strong internal consistency (Cronbach’s α > .91) and moderate test-retest reliability (r = .59) (Bova et al., 2012). The WPCS is a 5-item measure of human caring practices using a 7-point scale (Cronbach’s α = .90) (Brewer & Watson, 2015). The PaTAT consists of 25 items that evaluate trust in electronically delivered health services (Van Velsen et al., 2016; Velsen et al., 2017).

Adherence to ART was measured using a validated self-report scale widely used in HIV care (Wilson et al., 2020). HIV stigma was assessed using the revised 12-item HIV Stigma Scale, which uses a 4-point Likert scale and has demonstrated acceptable internal consistency (Cronbach’s α > .70) (Reinius et al., 2017). Data collection spanned from June 2019 to November 2020 and was designed to capture eight months before and after the onset of the COVID-19 pandemic, operationalized as March 15, 2020, when emergency public health orders were enacted in Colorado. Participants were purposively sampled from two affiliated ambulatory HIV clinics located within a quaternary children’s hospital and its university-based adult counterpart. Eligible participants were 18 to 30 years old, living with perinatally or situationally acquired HIV, English-speaking, and receiving or previously received care at one of the two sites. Although the study targeted this age range, a small number of individuals (n = 7) over age 30 with perinatally acquired HIV were included due to their participation in a long-term observational study and to capture insights related to their transition to adult HIV care. We excluded pregnant individuals due to the increased frequency of visits associated with perinatal care standards. Recruitment occurred via text, phone, email, and secure messaging through the electronic health record. Surveys were administered via REDCap (Harris et al., 2009) at the University of Colorado following electronic informed consent. The Colorado Multiple Institutional Review Board (COMIRB #20–2536) approved the study. Data analysis included chi-square, Fisher’s exact, and Wilcoxon rank sum tests, conducted using SPSS software (IBM, 2020).

## Results

Of the 281 youth with HIV (YWH) who were screened and approached for participation, 109 individuals (38.8%) provided informed consent, completed the electronic survey, and were included in the final sample for the chart abstraction. The average age of participants was 26.4 years (SD = 4.04); 60.6% identified as male, 1.8% as transgender female, and 0.9% as transgender male. Regarding race and ethnicity, 48.7% identified as Black, American Indian, or mixed race, and 73.4% identified as non-Hispanic or Latino. A total of 43 participants (39.4%) had perinatally acquired HIV. Seventy participants (64.2%) identified their primary HIV provider as a physician, while 38 (34.9%) received care from a nurse practitioner; one participant had missing provider information and was excluded from provider-level comparisons (Table 1).

Participant characteristics-including race, ethnicity, gender, and mode of HIV acquisition-did not differ significantly by provider type (Table 2). There were no significant differences in HIV viral load (p = 0.907; mean difference [95% CI]: 0.07 [–0.07, 0.22]) or engagement in care-defined as having at least one pre- and post-COVID visit, one HIV RNA PCR test, and all PCRs < 200 copies/μL-between provider types (49.0% among participants with physician providers vs. 44.0% among those with nurse practitioner providers; p = 0.703; φ = 0.017; Table 3) over the full 16-month study period (June 2019–November 2020). Participants who received care from nurse practitioners also had significantly higher mean CD4 absolute counts and CD4 percentages compared to those with physician providers (Table 4). The mean CD4 absolute count was 765.0 cells/μL for NP patients and 613.0 cells/μL for physician patients (p = 0.043; Cohen’s d = 0.451). The mean CD4 percentage was 35.5% among NP patients and 30.8% among physician patients (p = 0.039; Cohen’s d = 0.460), reflecting small to moderate effect sizes.

Notably participants who received care from a nurse practitioner had a higher mean number of total HIV care visits during that period (mean = 4.0) than those who received care from a physician (mean = 3.0; p = 0.019; Cohen’s d = 0.455) (Table 3). Overall, 59.0% of participants received their HIV care in person. In-person visits were more frequent among those with physician providers (78.0%) than those with nurse practitioner providers (22.0%; p = 0.010). Conversely, participants with nurse practitioner providers had significantly more telehealth visits (55.0%) than those with physician providers (45.0%; p = 0.002) (Table 2).

Among patient-reported outcomes, 23.0% of all participants scored in the high range on the patient-provider trust scale, defined as a total score of ≥ 49 (Bova et al., 2012) (Table 4). A greater proportion of nurse practitioner–treated participants achieved high trust scores (≥ 49) compared to those treated by physicians (65.8% vs. 42.9%; p = 0.038; φ = 0.200). Middle-range trust scores (40–48) were reported by 32.4% of participants, while low trust (< 40) was less common and did not differ significantly between groups (15.8% vs. 17.1%; p = 1.000; φ = 0.000) (Table 4). No significant differences were found in continuous trust scores across the HCR Trust Scale, Watson Patient Caring Score (WPCS), or Patient Trust Assessment Tool (PaTAT), supporting the overall pattern of greater high-trust ratings among NP-managed youth without mean differences across full trust scales (Table 4).

No significant differences were observed in medication adherence (73.0% for nurse practitioner patients vs. 87.0% for physician patients; p = 0.154), depressive symptoms (PHQ-4 ≥ 6: 63.2% vs. 62.9%; p = 1.000), psychological distress (K6 ≥ 5: 36.8% vs. 31.4%; p = 0.722), or HIV stigma (mean difference [95% CI]: 0.02 [–0.23, 0.28]; p = 0.855) between provider groups. (Table 4).

## Discussion

The results of this study suggest that nurse practitioners (NPs) and physicians provide comparable HIV care to youth across several critical clinical outcomes, including HIV viral load suppression, engagement in care, medication adherence, and HIV stigma. Demonstrating equivalency in these areas is an important contribution, especially in the context of a growing HIV provider shortage and geographic disparities in access to care (Armstrong, 2020; Bono et al., 2021; Department of Health and Human Services, 2024).

Ensuring that NPs provide care that meets the same clinical benchmarks as physicians supports their broader integration into the HIV workforce and offers a sustainable strategy for scaling youth-focused HIV services. At the same time, NPs demonstrated modest but meaningful advantages in specific domains. Participants cared for by NPs had significantly more visits, greater engagement in telehealth, and reported higher levels of trust in their providers. The difference in telehealth engagement, supported by a small-to-moderate effect size (φ = 0.245), underscores the potential for NP-led models to improve care flexibility and access for youth living with HIV. In addition, participants in NP care had higher CD4 absolute counts and CD4 percentages, with small-to-moderate effect sizes. While these immune function differences are multifactorial, they may reflect greater continuity, accessibility, or relational engagement in NP-managed care. This may also be related to sicker patients being assigned to physicians when they enter HIV care, however that was not a standard practice at either of these HIV clinical programs. These findings suggest that in some cases, NP care may not only be equivalent but beneficial across dimensions of access, immune health, and trust.

Trust emerged as a particularly important differentiator in this study and aligns with the Trust-Link Relational Transition Model (Blinded, 2025), which conceptualizes trust as a relational process that supports continuity and engagement in care. Youth receiving NP care were significantly more likely to report high levels of trust (≥ 49 on the HCR Trust Scale-R), while low trust scores were relatively uncommon and did not differ significantly between groups. The majority of remaining participants fell into a middle trust range (40–48), reported by 32.4% of the sample (Table 4). Prior research shows that trust mediates the relationship between health literacy and both retention and medication adherence in HIV care (Mgbako et al., 2022). Notably, every five-point increase on a 60-point trust scale is associated with a nearly 25% increase in care retention (Graham et al., 2015). Trust has also been described by patients as integral in quality care, particularly in people with HIV who may experience stigma and marginalization (Mgbako et al., 2022).

Consistent with the Trust-Link model’s emphasis on informed trust and reciprocal collaboration, higher trust among NP-managed youth may reflect relational practices that promote engagement and continuity rather than discipline-specific differences in clinical competence. These findings address an important evidence gap regarding the quality and characteristics of HIV care delivered by NPs to adolescents and young adults. Rather than being positioned merely as substitutes for physicians, NPs may offer unique strengths-including enhanced trust, flexibility in care delivery, and immune health outcomes-that support their value in HIV workforce planning. Future studies should build on these findings across larger, multi-site samples to further explore how NP-led care may promote health equity and continuity among youth with HIV, especially in underserved or high-need settings.

## Implications for Practice

### Expanding HIV Services Through NP Workforce

Given the high proportion of undiagnosed HIV and low testing rates among young people, findings from this study support targeted investment in a nurse practitioner (NP) HIV workforce focused on youth to expand access to testing, treatment, and linkage to care, including through the Ryan White HIV/AIDS Program (RWHAP). Although RWHAP serves more than 500,000 individuals nationally, only one in five patients is a youth with HIV, with similarly low representation across rural and non-rural settings (Klein et al., 2020). Notably, the percentage was roughly equal for rural (20.0%) and non-rural (22.0%) RWHAP providers (Klein et al., 2020). Limited access to youth-focused HIV testing and linkage, particularly in rural and southern regions, has been identified as a contributor to persistent geographic disparities in HIV incidence (Klein et al., 2020). Expanding NP-led HIV services for young people aligns with evidence supporting community- and school-based HIV prevention approaches and may strengthen linkage to care in underserved settings (Mavedzenge et al., 2014).

## School-Based Health Centers as a Strategic Platform

School-based health centers (SBHCs) represent a promising platform for NP-led HIV prevention and linkage to care. A recent CDC initiative implemented across 28 school districts reached approximately two million students and demonstrated the feasibility of integrating HIV testing and referral within school settings (Wilkins et al., 2022). Nationally, NPs provide the majority of preventive services in SBHCs, positioning them as key providers for youth-focused HIV care (Soleimanpuor S., 2023). Evidence from SBHC settings shows improved uptake of STI testing and other preventive services when interventions are delivered by NPs rather than computer-based models (Sharma et al., 2022), as well as benefits across reproductive and mental health outcomes (Arenson et al., 2019). Given the higher trust and telehealth engagement observed among youth receiving NP care in this study, integrating NP-led relational and telehealth approaches within SBHCs may enhance HIV testing, referral, and continuity of care.

### Community-Based NP Services for Youth with HIV

Beyond school settings, expanding community-based NP HIV services for youth may further improve prevention and engagement. Prior evidence indicates that NPs are more likely than physicians to deliver key HIV prevention services, including pre-exposure prophylaxis prescribing (Zhang et al., 2020). Systematic reviews of HIV care delivery models suggest comparable clinical outcomes across provider types, with advanced practice models offering a feasible approach for comprehensive HIV care delivery (Kimmel et al., 2016). Together with the current findings, this evidence supports the expansion of community-based NP HIV services as a strategy to improve access, trust, and continuity of care for youth with HIV.

## Limitations and Strengths

This study has several limitations. The sample size was relatively small and drawn from a single institution, which may limit generalizability. However, these findings provide a valuable foundation for future multi-site studies to further investigate differences in HIV care delivery models. Additionally, CD4 values were analyzed post hoc and not part of the original statistical plan, warranting cautious interpretation. Despite these limitations, this study has notable strengths. We assessed multiple patient-reported outcomes using validated instruments, including trust, adherence, and stigma-domains that are often underexplored in HIV care for adolescents and young adults (Mgbako et al., 2022; Reinius et al., 2017). Our evaluation of these psychosocial and engagement-related factors in YWH, a population often underrepresented in the literature, provides a unique contribution. Furthermore, our use of patient-reported outcome measures supports the advancement of patient-centered HIV care that includes shared decision-making and individualized care planning (Carfora et al., 2022). These elements of care delivery are especially critical to meet the developmental and relational needs of youth living with HIV (Opel, 2018).

## Conclusion

This study provides evidence that nurse practitioners deliver HIV care to adolescents and young adults that is comparable to physician care across key clinical outcomes, with additional strengths in trust, visit frequency, and telehealth engagement. These findings support the role of nurse practitioners as a critical component of a youth-focused HIV workforce at a time of persistent provider shortages and inequities in access to care. Higher trust among youth receiving NP care highlights the importance of relational approaches in sustaining engagement during a vulnerable developmental period. Expanding NP-led HIV care models may represent a feasible and effective strategy to enhance access, continuity, and patient-centered care for youth living with HIV. Future multi-site studies are warranted to further examine how NP-delivered care can be leveraged to advance equity and engagement across diverse HIV care settings.

## Supplementary Material

Supplementary Files

This is a list of supplementary files associated with this preprint. Click to download.

- SupplementalTable1.docx

## Figures and Tables

**Table 1:** Participant Demographics

Category	Subcategory	Total Sample (n = 109) n (%)
**Age**	Age (mean, SD)	26.4 (4.04)
**Age Group**	18–24	39 (35.8%)
	25–30	63 (57.8%)
	31 and older	7 (6.4%)
**Gender**	Female	41 (37.6%)
	Male	65 (59.6%)
	Trans female	2 (1.8%)
	Trans male	1 (0.9%)
**Race/Ethnicity** ^a^	American Indian	3 (2.8%)
	Black or African American	29 (26.6%)
	More than one race	9 (8.3%)
	Unknown	12 (11.0%)
	White or Caucasian	56 (51.4%)
	Hispanic or Latino	29 (26.6%)
	Not Hispanic or Latino	80 (73.4%)
**Education**	Some high school	11 (10.1%)
	HS graduate or some college	76 (69.7%)
	College degree or higher	22 (20.2%)
**HIV Acquisition**	Perinatally acquired	43 (39.4%)
	Situationally acquired	66 (60.6%)
**Transitioned Care**	Pre-transition (still in adolescent care)	37 (33.9%)
	Transitioned to adult care	63 (57.8%)
	In adult clinic, never transitioned	9 (8.3%)
**Current HIV Care Provider** ^b^	Physician	70 (64.2)
	Nurse practitioner	38 (34.9)

Note:

a Participants chose all races and ethnicities that applied to them.

b One participant stated they did not know their provider.

**Table 2:** Comparison of Clinical and Visit Characteristics by Provider Type

Variable	Level	Total n(%)	Physician	NP	p-value
**N** ^a^		109 (100%)	70 (64.2%)	38 (34.9%)	
**Gender**	Female or trans-female	43 (39.4%)	26 (37.1%)	16 (42.1%)	0.765
	Male or trans-male	66 (60.6%)	44 (62.9%)	22 (57.9%)	
**Race**	People of Color and White plus Hispanic	66 (61.1%)	41 (58.6%)	25 (67.6%)	0.483
	White and non-Hispanic	42 (38.9%)	29 (41.4%)	12 (32.4%)	
**Education**	HS graduate or higher	75 (68.8%)	51 (72.9%)	24 (63.2%)	0.409
	No school through some HS	34 (31.2%)	19 (27.1%)	14 (36.8%)	
**HIV Mode of Acquisition**	Perinatally acquired	43 (39.4%)	29 (41.4%)	14 (36.8%)	0.796
	Non-perinatally acquired	66 (60.6%)	41 (58.6%)	24 (63.2%)	
**Ever Transitioned from Adolescent to Adult Care**	Never transitioned to Adult Care	51 (46.8%)	29 (41.4%)	22 (57.9%)	0.151
	Transitioned to Adult Care	58 (53.2%)	41 (58.6%)	16 (42.1%)	
**Telehealth Use** ^b^	Yes	76 (70.4%)	43 (61.4%)	33 (86.8%)	0.011
**Total HIV Care Visits** ^c^	Median [IQR]		3.00 [2.00, 4.00]	4.00 [3.00, 5.00]	0.019
**Total Telehealth Visits** ^c^	Median [IQR]		0.00 [0.00, 1.00]	1.00 [0.00, 1.00]	0.002
**Total Hybrid Visits** ^d^	Median [IQR]		0.00 [0.00, 0.00]	0.00 [0.00, 1.00]	0.001

Note:

a Total study sample was 109 participants; however, one individual had missing provider type and was excluded from provider-level comparisons.

b Telehealth use is defined as having had at least one telehealth visit during the 16-month study period.

c Total HIV care visits, telehealth visits, and hybrid visits represent the number of each visit type per participant over the full study period.

d A hybrid visit was defined as an in-person visit in which one or more care team members (e.g., physician, social worker, or case manager) joined remotely via telehealth. Median values of 0.00 indicate that more than half of participants in that provider group did not experience that specific visit type.

**Table 3:** HIV Outcomes, Visits and Engagement by Provider Type

Measure	Level	NP (n = 38)	Physician (n = 70)	p-value	Effect Size
**CD4 absolute count (mean, SD)**		765.0 (365.2)	613.0 (306.1)	0.043	Cohen’s d = 0.451
**CD4 percentage (mean, SD)**		35.5 (10.3)	30.8 (10.1)	0.039	Cohen’s d = 0.460
**HIV RNA PCR < 200 copies/μL** ^a^	Yes	24 (66.7%)	45 (71.4%)	0.788	φ = 0.027
**Total HIV care visits (median [IQR])**		4.00 [3.00, 5.00]	3.00 [2.00, 4.00]	0.019	Cohen’s d = 0.455
**Engaged in care** ^b^	Yes	44.0%	49.0%	0.703	φ = 0.017

Note:

a All HIV RNA PCRs were in this range in the total 16 month study time period.

b Engagement in care was defined as having at least one HIV care visit both before and after March 15, 2020 (the COVID-19 emergency declaration in Colorado), at least one HIV RNA PCR test during the study period, and all HIV RNA PCR results < 200 copies/μL.

**Table 4:** Patient-Reported Outcomes by Provider Type

Measure	Level	NP (n = 38)	Physician (n = 70)	p-value	Effect Size	Total (n = 108)
Trust (HCR ≥ 49)	High trust	25 (65.8%)	30 (42.9%)	0.038	φ = 0.200	55 (50.9%)
Trust (HCR 40–48)	Middle trust	7 (18.4%)	28 (40.0%)	---	---	35 (32.4%)
Trust (HCR < 40)	Low trust	6 (15.8%)	12 (17.1%)	1.000	φ = 0.000	18 (16.7%)
Adherence	Good adherence	33 (86.8%)	51 (72.9%)	0.154	φ = 0.137	84 (77.8%)
Depression symptoms (PHQ-4 ≥ 6)	Depressed	24 (63.2%)	44 (62.9%)	1.000	φ = 0.000	68 (63.0%)
Psychological distress (K6 ≥ 5)	Moderate to severe	14 (36.8%)	22 (31.4%)	0.722	φ = 0.034	36 (33.3%)

Note: HCR trust score defined as “low” or “high” based on the cited literature. Cut off values for having depressive symptoms on the PhQ-4 and psychological distress on the K6 is used based on the cited literature.

## References

[1] Armstrong W. S. (2020). The Human Immunodeficiency Virus workforce in crisis: An urgent need to build the foundation required to End the Epidemic. Clinical Infectious Diseases, 72(9), 1627–1630. 10.1093/cid/ciaa302 32211784

[2] Blinded (2025). Manuscript

[3] Bono R. S., Dahman B., Sabik L. M., Yerkes L. E., Deng Y., Belgrave F. Z., Nixon D. E., Rhodes A. G., & Kimmel A. D. (2021). Human Immunodeficiency Virus-experienced clinician workforce capacity: Urban-rural disparities in the Southern United States. Clinical Infectious Diseases, 72(9), 1615–1622. 10.1093/cid/ciaa300 32211757 PMC8096280

[4] Bova C., Route P. S., Fennie K., Ettinger W., Manchester G. W., & Weinstein B. (2012). Measuring patient-provider trust in a primary care population: Refinement of the health care relationship trust scale. Research in Nursing and Health, 35(4), 397–408. 10.1002/nur.21484 22511461

[5] Brewer B. B., & Watson J. (2015). Evaluation of authentic human caring professional practices. The Journal of Nursing Administration, 45(12), 622–627. 10.1097/nna.0000000000000275 (J Nurs Adm) 26502069

[6] Budak J. Z., Sears D. A., Wood B. R., Spach D. H., Armstrong W. S., Dhanireddy S., Teherani A., & Schwartz B. S. (2021). Human Immunodeficiency Virus training pathways in residency: A national survey of curricula and outcomes. Clinical Infectious Diseases, 72(9), 1623–1626. 32211781 10.1093/cid/ciaa301

[7] Bureau of Labor Statistics, U. S. D. o. L. (2023). Occupational Outlook Handbook, Nurse Anesthetists, Nurse Midwives, and Nurse Practitioners. https://www.bls.gov/ooh/healthcare/nurse-anesthetists-nurse-midwives-and-nurse-practitioners.htm

[8] Carfora L., Foley C. M., Hagi-Diakou P., Lesty P. J., Sandstrom M. L., Ramsey I., & Kumar S. (2022). Patients’ experiences and perspectives of patient-reported outcome measures in clinical care: A systematic review and qualitative meta-synthesis. PLoS One, 17(4), e0267030. 10.1371/journal.pone.0267030 35446885 PMC9022863

[9] Centers for Disease Control and Prevention. (2023). Estimated HIV incidence and prevalence in the United States, 2017–2021. HIV Surveillance Supplemental Report, 28(3). http://www.cdc.gov/hiv/library/reports/hiv-surveillance.html

[10] Centers for Disease Control and Prevention. (2021). Estimated HIV incidence and prevalence in the United States 2015–2019. (HIV Surveillance Supplemental Report, Issue. https://www.cdc.gov/hiv/pdf/library/reports/surveillance/cdc-hiv-surveillance-supplemental-report-vol-26-1.pdf

[11] Dasgupta S., Tie Y., Beer L., Fagan J., & Weiser J. (2021). Barriers to HIV care by viral suppression status among US adults with HIV: Findings from the Centers for Disease Control and Prevention Medical Monitoring Project. Journal of the Association of Nurses in AIDS Care, 32(5), 561–568. 10.1097/jnc.0000000000000249 PMC8628483 33769329

[12] Department of Health and Human Services, H. S. R. a. A. (2024). Health Workforce Projections. https://data.hrsa.gov/topics/health-workforce/workforce-projections

[13] Farley J. E., Stewart J., Kub J., Cumpsty-Fowler C., Lowensen K., & Becker K. (2016). Development of The Johns Hopkins University School of Nursing Adult/Geriatric Primary Care Nurse Practitioner Program in HIV Prevention, Treatment, and Care. Journal of the Association of Nurses in AIDS Care, 27(3), 223–233. 10.1016/j.jana.2015.12.006 26852319

[14] Fields E. L., Louis-Jacques J., Kas-Osoka O., Holland-Hall C., Richardson L. P., Ott M., Leslie L. K., & Pitts S. A. B. (2024). Child health needs and the adolescent medicine workforce supply: 2020–2040. Pediatrics, 153(Suppl 2). 10.1542/peds.2023-063678D 38300009

[15] Gilman B., Bouchery E., Hogan P., Negrusa S., Trent-Adams S., & Cheever L. (2016). The HIV clinician workforce in the United States: Supply and demand projections from 2010 to 2015. HIV Specialist, 8(3), 2–9.

[16] Gilman B., B. E., Barrett K., Stalley S., Hargreaves M., Thomas C., Miller D., McCauley J,. (2013). HIV Clinician Workforce Study Final Report. (Report submitted to U.S. Department of Health and Human Services, Health Resources and Services Administration, HIV AIDS Bureau.

[17] Graham J. L., Shahani L., Grimes R. M., Hartman C., & Giordano T. P. (2015). The influence of trust in physicians and trust in the healthcare system on linkage, retention, and adherence to HIV care. AIDS Patient Care and STDs, 29(12), 661–667. 10.1089/apc.2015.0156 26669793 PMC4684652

[18] Harris P. A., Taylor R., Thielke R., Payne J., Gonzalez N., & Conde J. G. (2009). Research electronic data capture (REDCap)--a metadata-driven methodology and workflow process for providing translational research informatics support. Journal of Biomedical Informatics, 42(2), 377–381. 10.1016/j.jbi.2008.08.010 18929686 PMC2700030

[19] HHS. (2022, July 01, 2022 (exp. Date 8/31/2023)). What Is ending the HIV epidemic in the U.S.? Department of Health and Human Services. Retrieved September 12, 2022 from https://www.hiv.gov/federal-response/ending-the-hiv-epidemic/overview

[20] HRSA H. R. S. A. (2019). HIV/AIDS Bureau Performance Measures In Gap in Medical Visits (Medical Case Management) (pp. 2). Washington, D.C.: Ryan White HIV/AIDS Program.

[21] IBM. (2020). IBM SPSS Statistics for Windows. In (Version 27)

[22] K. G. (2023). 20 Fastest-growing occupations - Nurse practitioner is No. 1. Becker’s Hospital Review. https://www.beckershospitalreview.com/workforce/20-fastest-growing-occupations-nurse-practitioner-is-no-1.html

[23] Kates J., Dawson L., Horn T. H., Killelea A., McCann N. C., Crowley J. S., & Walensky R. P. (2021). Insurance coverage and financing landscape for HIV treatment and prevention in the USA. Lancet, 397(10279), 1127–1138. 10.1016/s0140-6736(21)00397-4 33617778

[24] Kelly M. S., Cataldi J. R., Schlaudecker E. P., Shah S. S., Vinci R. J., & Myers A. L. (2024). Child health needs and the pediatric infectious diseases workforce: 2020–2040. Pediatrics, 153(Supplement 2). 10.1542/peds.2023-063678N PMC10852198 38300015

[25] Kimmel A. D., Martin E. G., Galadima H., Bono R. S., Tehrani A. B., Cyrus J. W., Henderson M., Freedberg K. A., & Krist A. H. (2016). Clinical outcomes of HIV care delivery models in the US: A systematic review. AIDS Care-Psychological and Socio-Medical Aspects of Aids/HIV, 28(10), 1215–1222. 10.1080/09540121.2016.1178702 PMC4972662 27177151

[26] Klein P. W., Geiger T., Chavis N. S., Cohen S. M., Ofori A. B., Umali K. T., & Hauck H. (2020). The Health Resources and Services Administration’s Ryan white HIV/AIDS program in rural areas of the United States: geographic distribution, provider characteristics, and clinical outcomes. PLoS One, 15(3), e0230121. https://journals.plos.org/plosone/article/file?id=10.1371/journal.pone.0230121&type=printable 32203556 10.1371/journal.pone.0230121 PMC7089565

[27] Mavedzenge S. N., Luecke E., & Ross D. A. (2014). Effective approaches for programming to reduce adolescent vulnerability to HIV infection, HIV risk, and HIV-related morbidity and mortality: A systematic review of systematic reviews. JAIDS Journal of Acquired Immune Deficiency Syndromes, 66. https://journals.lww.com/jaids/fulltext/2014/07011/effective_approaches_for_programming_to_reduce.3.aspx 10.1097/QAI.0000000000000178 24918591

[28] Mgbako O., Conard R., Mellins C. A., Dacus J. d., & Remien R. H. (2022). A systematic review of factors critical for HIV health literacy, ART adherence and retention in care in the U.S. for racial and ethnic minorities. AIDS and Behavior, 26(11), 3480–3493. 10.1007/s10461-022-03680-y 35445996 PMC9550694

[29] Mustanski B., Moskowitz D. A., Moran K. O., Rendina H. J., Newcomb M. E., & Macapagal K. (2020). Factors associated with HIV testing in teenage men who have sex with men. Pediatrics, 145(3). 10.1542/peds.2019-2322 PMC7049943 32047100

[30] Opel D. J. (2018). A 4-Step framework for shared decision-making in pediatrics. Pediatrics, 142(Suppl 3), S149–S156. 10.1542/peds.2018-0516E 30385621

[31] Reinius M., Wettergren L., Wiklander M., Svedhem V., Ekstrom A. M., & Eriksson L. E. (2017). Development of a 12-item short version of the HIV stigma scale. Health and Quality of Life Outcomes, 15(1), 115. 10.1186/s12955-017-0691-z 28558805 PMC5450123

[32] Schafer K. R., Albrecht H., Dillingham R., Hogg R. S., Jaworsky D., Kasper K., Loutfy M., MacKenzie L. J., McManus K. A., Oursler K. A. K., Rhodes S. D., Samji H., Skinner S., Sun C. J., Weissman S., Ohl M. E., & on behalf of the North American Rural, H. I. V. W. G. (2017). The continuum of HIV care in rural communities in the United States and Canada: What Is known and future research directions. JAIDS Journal of Acquired Immune Deficiency Syndromes, 75(1). https://journals.lww.com/jaids/fulltext/2017/05010/the_continuum_of_hiv_care_in_rural_communities_in.6.aspx 10.1097/QAI.0000000000001329 PMC6169533 28225437

[33] Sharma A., Mitchell S. G., Nordeck C. D., Schwartz R. P., Dusek K., O’Grady K. E., & Gryczynski J. (2022). Sexually transmitted infection testing after brief intervention for risk behaviors in school-based health centers. Journal of Adolescent Health, 70(4), 577–583. 10.1016/j.jadohealth.2021.11.013 PMC13382876 35078735

[34] Soleimanpuor S., C. K., Christensen J., Ng S., Yang J., Saphir M., Geierstanger S., Even M., Brey L.,. (2023). Findings from the 2022 National Census of School-based Health Centers. S.-B. H. Alliance.

[35] Stevens L. C., Webb A. A., Davis S., Corless I., & Portillo C. (2008). HIV care provider shortages highlighted in national meeting. Journal of the Association of Nurses in AIDS Care, 19(6), 412–414. 10.1016/j.jana.2008.09.005 19007717

[36] Van Velsen L., Wildevuur S., Flierman I., Van Schooten B., Tabak M., & Hermens H. (2016). Trust in telemedicine portals for rehabilitation care: an exploratory focus group study with patients and healthcare professionals. BMC Medical Informatics and Decision Making, 16, 11. 10.1186/s12911-016-0250-2 26818611 PMC4728819

[37] Velsen L. V., Tabak M., & Hermens H. (2017). Measuring patient trust in telemedicine services: Development of a survey instrument and its validation for an anticoagulation web-service. International Journal of Medical Informatics, 97, 52–58. 10.1016/j.ijmedinf.2016.09.009 27919395

[38] Weiser J., Beer L., West B. T., Duke C. C., Gremel G. W., & Skarbinski J. (2016). Qualifications, demographics, satisfaction, and future capacity of the HIV care provider workforce in the United States, 2013–2014. Clinical Infectious Diseases, 63(7), 966–975. https://www.ncbi.nlm.nih.gov/pmc/articles/PMC5058419/pdf/nihms816328.pdf 27358352 10.1093/cid/ciw442 PMC5058419

[39] Weiser J., Chen G., Beer L., Boccher-Lattimore D., Armstrong W., Kurth A., & Shouse R. L. (2019). Sustaining the HIV care provider workforce: Medical Monitoring Project HIV Provider Survey, 2013–2014. Health Serv Res, 54(5), 1065–1074. 10.1111/1475-6773.13192 31264205 PMC6736917

[40] Weiser J., Tie Y., Crim S. M., Riedel D. J., Shouse R. L., & Dasgupta S. (2024). Do HIV care outcomes differ by provider type? JAIDS Journal of Acquired Immune Deficiency Syndromes, 96(2), 180–189. 10.1097/QAI.0000000000003410 38465906 PMC11215811

[41] Wilkins N. J., Rasberry C., Liddon N., Szucs L. E., Johns M., Leonard S., Goss S. J., & Oglesby H. (2022). Addressing HIV/sexually transmitted diseases and pregnancy prevention through schools: An approach for strengthening education, health services, and school environments that promote adolescent sexual health and well-being. Journal of Adolescent Health, 70(4), 540–549. 10.1016/j.jadohealth.2021.05.017 PMC9260911 35305791

[42] Wilson I. B., Landon B. E., Hirschhorn L. R., McInnes K., Ding L., Marsden P. V., & Cleary P. D. (2005). Quality of HIV care provided by nurse practitioners, physician assistants, and physicians. Annals of Internal Medicine, 143(10), 729–736. 16287794 10.7326/0003-4819-143-10-200511150-00010

[43] Wilson I. B., Tie Y., Padilla M., Rogers W. H., & Beer L. (2020). Performance of a short, self-report adherence scale in a probability sample of persons using HIV antiretroviral therapy in the United States. AIDS, 34(15), 2239–2247. 10.1097/QAD.0000000000002689 32932340 PMC7674252

[44] Zapata J. P., Queiroz A., Rodriguez-Diaz C. E., & Mustanski B. (2024). Factors Associated with HIV Testing Among Spanish and English-Speaking Latino Adolescents Aged 13–18. AIDS and Behavior, 28(1), 343–356. 10.1007/s10461-023-04206-w 37848599 PMC12421724

[45] Zhang C., Mitchell W., Xue Y., LeBlanc N., & Liu Y. (2020). Understanding the role of nurse practitioners, physician assistants and other nursing staff in HIV pre-exposure prophylaxis care in the United States: a systematic review and meta-analysis. BMC Nursing, 19, 1–9. 33292201 10.1186/s12912-020-00503-0 PMC7724856

